# Adult-onset Still’s disease as the first manifestation of acute myeloid leukemia: a case report

**DOI:** 10.1097/MS9.0000000000000256

**Published:** 2023-02-17

**Authors:** Ranim Ibrahim, Tasneem Drie, Zienab Shahada, Hayat Al Halabi, Maysoun Kudsi

**Affiliations:** a Department of Rheumatology, Al-Mouwasat University Hospital; b Departmen of Rheumatology, Damascus University/Syrian Private University, Damascus, Syria

**Keywords:** adult-onset Still’s disease, AOSD-associated leukemia, case report, leukemic arthritis, paraneoplastic syndrome

## Abstract

**Introduction and importance:**

The association between adult-onset Still’s disease (AOSD) and malignancy has previously been observed. However, only a limited number of cases described a combination of AOSD and leukemia, none of which reported AOSD-related symptoms as the first manifestation of acute myeloid leukemia (AML). This presentation might represent a paraneoplastic syndrome or leukemic arthritis mimicking AOSD.

**Case presentation:**

Here the authors report a case of a 23-year-old female who fulfilled the Yamaguchi criteria for an AOSD diagnosis. She presented with complaints of polyarthritis, sore throat, and daily fever spikes with the appearance of a nonpruritic maculopapular salmon-colored rash. Her laboratory work showed marked pancytopenia, which led to a bone marrow examination and an AML diagnosis. The patient started receiving chemotherapy with considerable improvement in the AOSD-related symptoms.

**Clinical discussion:**

Patients with underlying malignancies could present with systemic features compatible with AOSD, which necessitates excluding malignancy in any patient with this presentation, specifically in light of some warning signs like pancytopenia.

**Conclusion:**

This case interprets a rare association between AOSD and AML. In addition, it highlights how crucial it is to be aware of the signs that should warn the clinician of a possible underlying malignancy in any patient presenting with AOSD-related symptoms.

Highlights

- Patients with underlying malignancies could present with adult-onset Still’s disease (AOSD) symptoms.
- Clinicians should be aware of the warning signs in AOSD patients.
- Leukemic arthritis could mimic AOSD.
- AOSD-associated leukemia might represent a paraneoplastic syndrome.

## Introduction

First coined by EG Bytwars in 1971, the term ‘adult-onset Still’s disease’ (AOSD) is used to refer to a rare systemic condition categorized as a multigenic autoinflammatory disorder^1^. It primarily affects young patients between 16 and 35 years. Rarely, a delayed onset after the age of 50 can also occur^2^.

There is no specific test for the diagnosis of AOSD. However, there are several proposed classification criteria sets, of which the Yamaguchi criteria are the most commonly used ones (Table 1). To consider the diagnosis of AOSD, five or more criteria (two or more majors) are required^3^.

**Table 1:** Yamaguchi classification criteria for adult-onset Still’s disease (AOSD)

Major criteria	1. Fever of at least 39°C for at least 2 weeks 2. Arthralgia or arthritis for at least 2 weeks. 3. Nonprutitic salmon-colored rash on trunk/extremeties. 4. Granulocytic leukocytosis (10 000/µl or greater)
Minor criteria	1. Sore throat 2. Lymphadenopathy, hepatomegaly, or splenomegaly 3. Abnormal liver function tests 4. Negative tests for antinuclear antibody (ANA) and rheumatoid factor (RF)
Exclusion criteria	Infections Malignancies (mainly malignant lymphoma) Other rheumatic disease (mainly systemic vasculitides)

Even though the association between AOSD and malignancy has previously been observed, the relationship underlying this association is not entirely understood^4^. Here we present the case of a young female diagnosed with acute myeloid leukemia (AML) masquerading as AOSD. This presentation might represent a paraneoplastic syndrome or leukemic arthritis mimicking AOSD.

This case report has been reported in line with the SCARE 2020 criteria^5^.

## Case presentation

**Figure 1:**
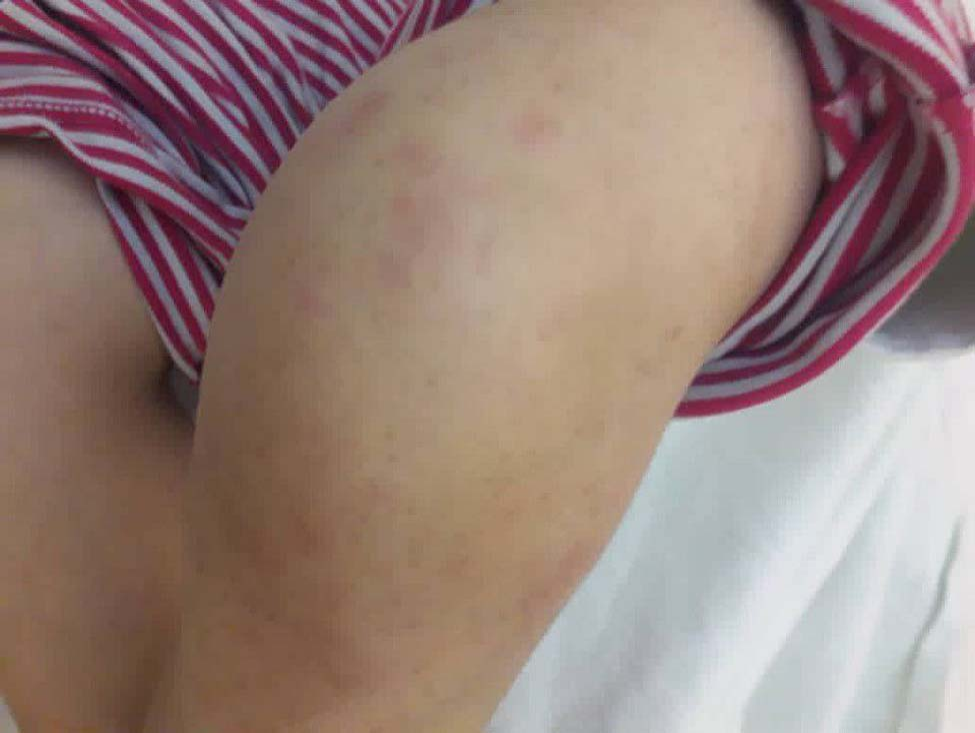
Shows the left swollen knee joint as well as the maculopapular salmoncolored rash.

A 23-year-old female presented to the rheumatology clinic with a 2-month complaint of symmetric polyarthritis involving the elbows, wrists, knees, and ankles, accompanied by a daily fever and significant weight loss. She reported that her symptoms were first associated with a sore throat, which had improved about 2 weeks before the presentation. She had no past morbidities or prior surgeries and did not smoke tobacco or drink alcohol. Her family history revealed that her younger brother was diagnosed with neuroblastoma at the age of four. She had received paracetamol for the past 2 months before the presentation and reported a resolution of her fever upon taking the medicine but no improvement in her pain. On examination, her heart rate was 117 beats per minute, her respiratory rate was 17 breaths per minute, and her blood pressure was 100/70 mm Hg. The patient was pale, irritable, and looked in pain. Her cardiac and pulmonary examinations were within normal limits. Her joint examination was positive for swelling and tenderness in her wrists, knees, and ankles. There was no lymphadenopathy, hepatomegaly, or splenomegaly. She was admitted to the rheumatology department for observation and further investigation. Her temperature chart showed a daily fever spike twice a day, with peaks reaching 40°C. On the second day of the admission, she developed a nonpruritic maculopapular salmon-colored rash during the febrile episodes (Fig. 1). The rash appeared mainly on the patient’s legs and upper extremities and was disappearing as the fever resolved.

Laboratory data at the time of presentation are summarized in Table 2. It showed marked pancytopenia with high inflammatory markers, high ferritin, and low albumin. Antinuclear antibody, rheumatoid factor, anti-Cyclic Citrullinated Peptide, hepatitis B surface antigen, antihepatitis C virus, parvovirus antibodies, and blood cultures were all negative. The glycosylated ferritin level could not be tasted since it was not available. Computed tomography scan of the chest, abdomen, and pelvis was done and did not show any findings suggesting a potential malignancy. The diagnosis of AOSD was considered appropriate because the patient fulfilled the Yamaguchi criteria. However, the presence of pancytopenia made it crucial to perform a bone marrow examination to exclude other disorders, including macrophage activation syndrome. It revealed a hypercellular marrow with an excess of immature blasts but no hemophagocytosis. The diagnosis of acute myelomonocytic leukemia (FAB classification M4) was made according to the immunophenotyping. A cytogenetic study was performed, and a translocation between chromosomes 6 and 9 with breakpoints at p22 and q34, respectively, was found. The patient started receiving chemotherapy with considerable improvement in the AOSD-related symptoms.

**Table 2:** Laboratory values at presentation

Laboratory parameter	Patient laboratory value at presentation	Reference range
Leukocyte count	2900/mm^3^	4500–11 000/mm^3^
Segmented neutrophils	66%	54–62%
Hemoglobin	6.5 g/dl	12.0–16.0 g/dl
Platelet count	100 000/mm^3^	150 000–400 000/mm^3^
Prothrombin time	12 s	11–15 s
Partial thromboplastine time	40.5 s	25–40 s
Erythrocyte sedimentation rate (ESR)	98 mm/h	0–20 mm/h
C- reactive protein (CRP)	34 mg/dl	Up to 0.5 mg/dl
Albumin	2.8 g/dl	3.5–5.5 g/dl
Ferritin	5000 ng/ml	12–150 ng/ml
Alanine aminotransferase (ALT)	10 U/l	10–40 U/l
Aspartate aminotransferase (AST)	13 U/l	12–38 U/l
Lactate dehydrogenase (LDH)	322 U/l	140–280 U/l
Creatinine	0.6 g/dl	0.6–1.2 g/dl
Uric acid	3.6 mg/dl	3–6 mg/dl

## Discussion

The association between AOSD and malignancy has previously been observed.

A solid tumor was diagnosed in half of the reported cases, with breast, lung, and thyroid cancers being the most frequent types^6^–^8^. The rest of the patients were diagnosed with a hematopoietic malignancy, prominently lymphoma^9^, with two cases of chronic myelogenous leukemia^10^,^11^, two cases of acute lymphoblastic leukemia^4^,^12^, and one case of myelodysplastic syndrome with later progression to AML^13^. These cases showed that the diagnosis of malignancy could precede, concur with, or follow AOSD. However, it followed the presentation of AOSD-related symptoms in the vast majority of the cases, with a median interval of nine months. To our knowledge, this is the first reported case of concurrent diagnoses of AOSD and AML.

AOSD-associated leukemia is rare and its cause remains unclear. The temporal relationship between these two entities plays a significant role in predicting the etiology underlying this association. Nakagawa and Sugawara published two cases of AOSD-associated leukemia, in which the diagnosis of AOSD preceded the diagnosis of leukemia by 2 and 6 years, respectively.

They both suggested that the cyclosporine used in AOSD treatment might have induced the development of leukemia^10^,^13^. We report concurrent diagnoses of AOSD and AML with marked improvement in the AOSD-related symptoms after chemotherapy. This presentation could represent a misinterpretation of leukemia’s symptoms and signs as AOSD. Leukemic cells can infiltrate into synovial tissue, causing symptoms and signs of arthritis. It has been widely observed that leukemic arthritis could mimic Still’s disease in children with acute lymphoblastic leukemia^14^. However, it is far less common in adults and more likely to present as seronegative reactive arthritis or rheumatoid arthritis rather than the AOSD-like disease^15^,^16^. Another possible explanation for this presentation is that it represents a paraneoplastic syndrome. Nevertheless, AOSD has not been listed as a true paraneoplasia as there was not enough evidence in the previously reported cases^4^.

Most of the AOSD classification criteria share the requirement of excluding malignancy before the final diagnosis. However, there are no obvious guidelines regarding how strict the application of this exclusion criterion should be. Hofheinz *et al*.^4^ published a review on AOSD-associated malignancy and identified several signs that should warn the clinician of a possible underlying malignancy in any patient presenting with AOSD, including the first presentation at a higher age than usual, atypical features of the skin rash, high levels of lactate dehydrogenase, and high concentrations of the soluble interleukin-2 receptor. The presence of pancytopenia, in our case, oriented us toward a bone marrow examination and an AML diagnosis. This finding may also suggest an accompanying macrophage activation syndrome, which can affect about 12–15% of AOSD patients^17^,^18^. However, this diagnosis was ruled out since it usually presents in severely ill patients with a number of specific features, such as unremitting fever, organomegaly, coagulopathy, hypertriglyceridemia, hypofibrinogenemia (indicated by a normal or low erythrocyte sedimentation rate), and the presence of hemophagocytosis in the bone marrow examination^18^.

Our approach was limited by the unavailability of both the cytokine tests and the glycosylated ferritin level test, which is a more specific marker for AOSD than serum ferritin^19^

## Conclusion

We report a case in which a presumptive diagnosis of AOSD was made with simultaneous detection of AML and demonstrated the significance of further investigation in any patient with AOSD-related symptoms in order to exclude any underlying malignancy, specifically in light of some warning signs like pancytopenia.

## Ethical approval

None.

## Consent

Written informed consent was obtained from the patient for the publication of this case report and accompanying images. A copy of the written consent is available for review by the Editor-in-Chief of this journal on request.

## Sources of funding

None.

## Author contribution

R.I.: literature review, manuscript writing and editing, review and approval of the final manuscript. T.D.: obtaining informed written consent, clinical follow up, manuscript writing, and approval of the final manuscript. Z.S. and H.A.: manuscript writing and approval of the final manuscript. M.K.: mentor and approval of the final manuscript.

## Conflicts of interest disclosure

All authors declare no conflict of interest.

## Research registration unique identifying number (UIN)

This case report do not detail a new surgical technique or new equipment/technology, this registration was not required.

## Guarantor

Ranim Ibrahim.

## Provenance and peer review

Not commissioned, externally peer-reviewed.
